# Medical Society of the County of Erie

**Published:** 1885-11

**Authors:** Edward Clark

**Affiliations:** Sec’y.


### Medical Society of the County of Erie.—Special Meeting.

A special meeting of the Medical Society of the County of Erie was held at the Y. M. C. A. building, Wednesday evening, Oct. 21, 1885, to take action on the death of Dr. Charles A. McBeth.

The meeting was called to order by the President of the Society, Dr. J. B. Andrews, who briefly stated the object for which the Society had met.

Dr. Fowler moved that the chair appoint a committee of three to prepare suitable resolutions, which was unanimously carried.

The President appointed as such committee, Drs. Fowler, Crego and Putman, who presented the following, which was unanimously adopted, viz.:

"As the Erie County Medical Society has assembled out of respect to the memory of Charles A. McBeth, M. D., we desire to give expression to our sense of loss at his untimely death. Though in practice but a few years, he had met with marked success. By untiring efforts and by persistent devotion to his profession, he had won the confidence and affection of a large circle. His reputation was that of a loyal friend and a man of frank and generous nature. It is sad to record the death of a young man whose natural abilities gave such great promise of future success. While we mourn our loss, we bow acknowledgment to the Great Physician who doeth all things well.

*Resolved*, That this memorial be spread upon the minutes of this Society, and a copy forwarded to his family.

*Resolved*, That this Society attend his funeral in a body."

Remarks in eulogy of the deceased were made by Drs. Fowler and Warren.

The President appointed Dr. Fowler a committee of one to prepare a memorial of Dr. McBeth to be presented to the Society at its meeting in January. On motion, the Society adjourned.

Edward Clark, M. D., Sec'y.

